# Ultrasonography for disorders of sex development in pediatrics

**DOI:** 10.3389/fped.2025.1506996

**Published:** 2025-03-24

**Authors:** Yuting Wu, Anqi Tao, Jigang Jing, Hua Zhuang

**Affiliations:** Department of Ultrasonography, West China Hospital of Sichuan University, Chengdu, China

**Keywords:** ultrasonography, disorders of sex development, ovotesticular DSD, 46, XY DSD, 46, XX DSD, chromosomal DSD

## Abstract

**Objective:**

This study aimed to evaluate the clinical value of ultrasonography in the management of disorders of sex development (DSDs).

**Methods:**

Ultrasonographic appearance and clinical data of 82 cases with DSD were reviewed retrospectively.

**Results:**

In total, there were 54 cases with the male phenotype and 28 cases with the female phenotype. All 12 cases with ovotesticular DSD were confirmed by surgery or pathological examination. Furthermore, 2 of 12 cases with ovotesticular DSD were misdiagnosed by ultrasonography.

**Conclusions:**

Ultrasonography can not only evaluate the internal sex organs in pediatric patients, but also estimate the type, location, size, and morphology of the gonads, which provides important imaging evidence for clinical diagnosis and treatment.

## Introduction

Disorders of sex development (DSDs) are congenital anomalies showing sex discordance in sex chromosomes, gonads, or sex hormones. According to the Chicago Consensus of 2006 (1), these disorders can be further subdivided into 46, XY DSD (disorders of gonadal or testicular development and impaired androgen synthesis or action), 46, XX DSD (disorders of gonadal or ovarian development and androgen excess), and chromosomal DSD (numerical sex chromosome anomalies). Patients with DSD may have different forms of sex chromosome abnormalities, abnormal gonadal development, and sex hormone synthesis and function disorders, forming three levels of sex mismatch, namely, gonadal sex and sex chromosome mismatch, internal and external reproductive organ development and gonadal sex mismatch, and sex hormone levels and gonadal sex mismatch (2). Because the condition is usually complex and there are various clinical manifestations, diagnosis and treatment are typically difficult. Ultrasonography (US) can distinctly show the presence, location, size, and morphological structure of the gonads (3). Compared with other imaging modalities, ultrasonography is a simple, inexpensive, radiation-free, and repeatable operation in pediatrics.

## Materials and methods

### Study subjects

From August 2010 to May 2024, 82 pediatric patients with DSD were diagnosed at the West China Hospital of Sichuan University, with 54 cases with the male phenotype and 28 cases with the female phenotype. These patients were aged 18 days to 18 years, with an average age of 5.44 ± 5.66 years. The principal symptoms included abnormal external genital morphology, a groin mass, amenorrhea, periodic “hematuria”, and obesity. All the patients underwent sex chromosome and blood hormone level examinations. Moreover, 30 cases underwent surgical procedures or exploratory laparotomy, 15 of which underwent surgical resection or biopsy for pathological examination.

### Equipment and methods

The Aixplorer US system (SuperSonic Imagine, France) with a convex transducer (C6-1) and linear convex transducers (L10-2 and L15-4) was used. During the examination, patients were in a supine position with a full bladder. Ultrasonography was conducted to explore the lower abdomen, inguinal region, and perineum of the patients to determine the presence of a uterus, ovaries, ovotestes, testicles, prostate, or vagina and to observe their shape, size, and structure. If necessary, ultrasonography was also used to detect whether there were enlarged adrenal glands in the adrenal regions. All examinations were performed by sonographers who had at least 5 years of experience.

### Statistics

SPSS software (version 25.0, SPSS Inc., Chicago, IL, USA) was used for statistical data processing and analysis. Enumeration data are presented as frequency and rate, and the measurement data are presented as mean ± SD.

## Results

The phenotypes of the 82 patients with DSD are shown in Table 1. In total, 12 cases were confirmed to be ovotesticular DSD by surgery or pathological examination, 2 (16.7%) of which were misdiagnosed by ultrasonography. In one case, testicles were found by ultrasonography, but ovarian stroma and follicles and a curved fine tubular structure of the testis were found in a pathological examination. In the other case, dysplastic gonads with sex cord-like interstitial and follicular structures were confirmed by pathological examination, but ultrasonography did not find them. In total, 15 cases underwent surgical resection or biopsy for a pathological examination, and in 13/15 (86.7%) cases, the ultrasonography results were consistent with the pathological examination results. The ultrasonic manifestations of 82 patients are shown in Table 2. Three of the five cases diagnosed with chromosomal DSD had Klinefelter syndrome of 47, XXY, and the other two cases were chimeras.

**Table 1:** The phenotypes and karyotypes of the 82 patients with DSD.

Category	*N*	Male phenotype	Female phenotype
Chromosomal DSD	5	5	0
46, XY DSD	58	42	16
46, XX DSD	19	7	12

*N* is the number of cases.

**Table 2:** The ultrasound manifestations of the 82 patients.

Category	Uterus/ovary		Testicle/ovotestis				
	Present	Absent	Inguen	Abdomen	Scrotum	Labium majus pudendi	Absent
Chromosomal DSD	0	5	2	0	3	0	0
46, XY DSD	4	53	23	3	27	2	3
46, XX DSD	15	4	6	1	2	0	10

## Discussion

Genders can be distinguished according to the sex chromosome karyotype, gonadal structure, external genital organ shape, and secondary sexual characteristics. According to the morphology of the vulva, patients with DSD can partly present as male phenotype and female phenotype. The male phenotype shows different degrees of short penile, curvature, hypospadias, scrotal emptiness, and scrotal schisis, such as labia majora or a groin mass. The female phenotype shows different degrees of clitoral hypertrophy, such as a tiny penis, and a large labia, such as a scrotum or a groin mass. In our study, there were 54 cases with the male phenotype and 28 cases with the female phenotype.

**Figure 1:**
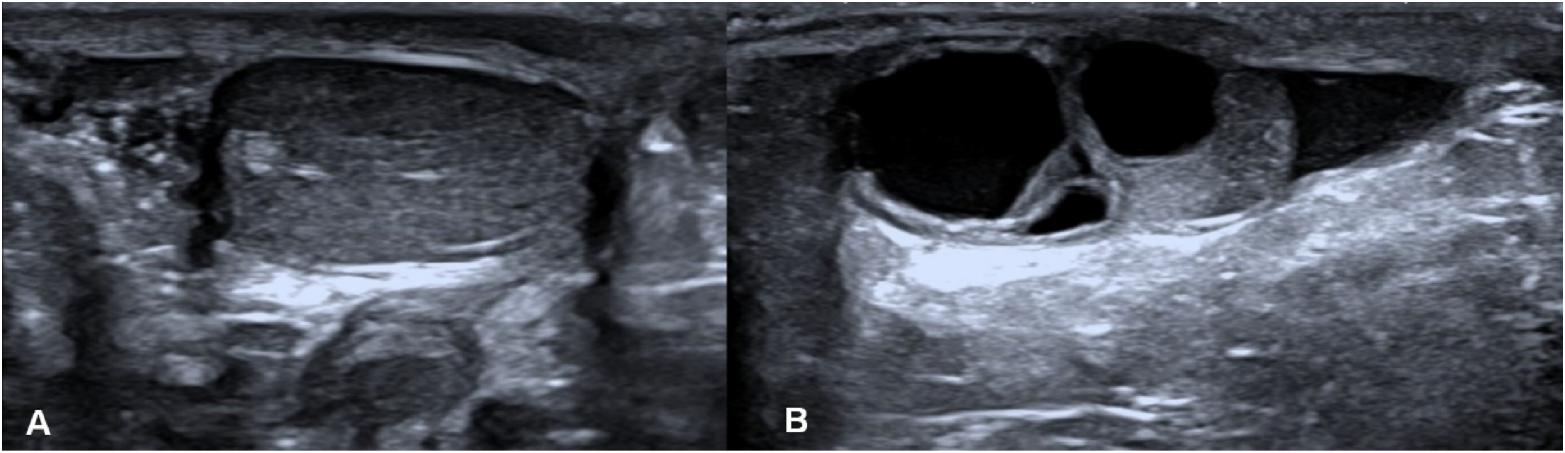
Lateral type of gonadal malformation. **(A)** The testicle is on the right, and **(B)** the ovary is on the left.

**Figure 2:**
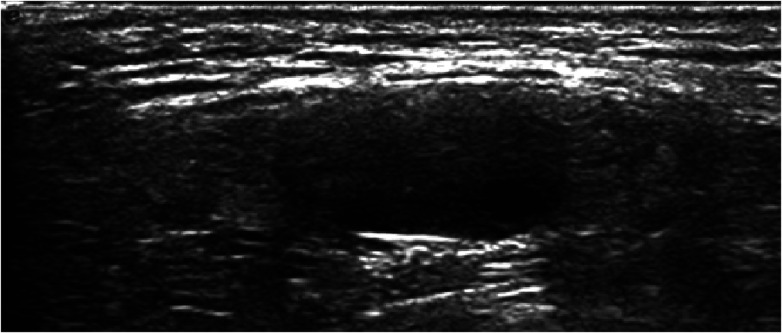
Ovotestis on ultrasonography.

Ovotesticular DSD occurs in all three DSD categories, but 65%–95% of cases can be classified as 46, XX DSD (4). In this study, 8 of the 12 (67%) ovotesticular DSD cases were of the 46, XX karyotype. This refers to both male and female gonadal tissue in the same body. Depending on the location of the two gonads, gonadal malformation has three forms: (1) bilateral type, with an ovotestis present on both sides; (2) unilateral type, with an ovotestis present on one side and a testicle or ovary present on the other side (Figure 1); (3) lateral type, with an ovary present on one side and a testicle present on the other side. The lateral type is the rarest, and the unilateral type with an ovary present on the other side is the most common (5). Ovotesticular DSD can be diagnosed if ultrasonography reveals bilateral gonadal inconsistencies or bilateral ovotestes, regardless of karyotype 46, XX, 46, XY, or other chimeras. An ovotestis is defined as the coexistence of ovarian and testicular tissue in one gonad. Ultrasonography can find ovotestes if there is a clear boundary and a complete envelope, with ovarian tissue and testicular tissue located end to end at two poles and a clear boundary between them. A small follicle-like echo can be seen on the ovarian side, and the echo on the testicular side is uniform (Figure 2). In this study, ultrasonography misdiagnosed two cases. In one case, a small follicle-like echo of the ovotestes was not obvious, and the ovotestes presented like testicles on ultrasonography. In the other case, ultrasonography failed to find the gonads because of gonadal dysplasia.

Because the discordance between the chromosomal, gonadal, and phenotypic sex in children with DSD determines the course of management and the type of treatment considered, imaging examinations play a major role in the assessment of the type, location, size, and morphology of the gonads in children's anatomy, especially in the surgical decision-making process. Ultrasonography is the most widely used imaging technique for internal sex organs, as it is easily accessible and does not require the use of radiation or contrast material (6).

To improve ultrasonographic diagnostic accuracy for DSD, operators should fully understand the embryonic development of the sex organs and genetic epidemiology and have basic pathophysiological knowledge of amphoteric malformations, such as the location of the gonads and the path of descent. The ovaries are commonly located behind the bladder in the pelvic cavity. An ovotestis can appear in the pelvic cavity, the groin region, the scrotum, or the labium majus pudenda. Cryptorchidism is usually found in the inguinal region. When suspecting 46, XX DSD, operators should scan the bilateral adrenal area and observe whether there are enlarged adrenal glands. Congenital adrenal cortical hyperplasia typically shows bilateral diffuse enlargement and elongation of the adrenal glands, with uniform and central linear high echoes and an occasional limited, nodular low echo on ultrasonography. This disorder should be distinguished from primary and secondary adrenal tumors, functional adrenal hyperplasia, and adrenal adenoma. The uterus, ovaries, and vagina of patients with DSD are frequently poorly displayed on conventional ultrasonography due to dysplasia. Ultrasonography images can be improved when children’s perineum is examined with high-frequency probes and adults are examined with rectal probes.

Previous research studies have compared different imaging modalities in the assessment of DSD. Mansour et al. showed that the accuracy of multi-approach ultrasound was 89.8% compared to 85.7% for magnetic resonance imaging (MRI) (7, 8). Another study found that, for the evaluation of the gonads, MRI was marginally more sensitive than US, and for internal genital structures, both modalities were found to be equally sensitive and specific with no false positive results (8). Professor Nasir Abdulllah Al Jurayyan studied the difference between ultrasound, genitogram, and MRI in patients with ambiguous genitalia. The sensitivity of ultrasound was 89.5%, while its specificity reached 100%. Retrograde genitogram was more invasive and less sensitive, as the rate of determining the presence of a uterus and/or a vagina was 84.2%. However, the specificity of MRI reached up to 100% and could provide detailed internal structures (9). In our study, we failed to compare ultrasonography to other imaging modalities in the assessment of DSD because other imaging data were not fully collected. However, we proved that ultrasonography has a high level of consistency with pathological examination results.

It has been reported in the literature that patients with DSD with a Y chromosome or Y fragment have a higher probability of gonadal tumors (10–12), while the opposite is true for patients with 46, XX DSD, as they have a very low incidence of tumors. This is due to patients with 46, XY DSD having a gene locus related to the occurrence of gonadal tumors on the Y chromosome (13). Therefore, patients with 46, XY DSD require an early diagnosis and surgical resection. Patients with 46, XX DSD can retain a reasonably developed gonad. Ultrasonography has superior accuracy for the diagnosis and differential diagnosis of pediatric DSD and can also be used as the first choice for preoperative/intraoperative examination and postoperative follow-up.

## Data Availability

The raw data supporting the conclusions of this article will be made available by the authors, without undue reservation.
